# Hepatic steatosis in n-3 fatty acid depleted mice: focus on metabolic alterations related to tissue fatty acid composition

**DOI:** 10.1186/1472-6793-8-21

**Published:** 2008-12-01

**Authors:** BD Pachikian, AM Neyrinck, PD Cani, L Portois, L Deldicque, FC De Backer, LB Bindels, FM Sohet, WJ Malaisse, M Francaux, YA Carpentier, NM Delzenne

**Affiliations:** 1 Unit of Pharmacokinetics, Metabolism, Nutrition and Toxicology, Université catholique de Louvain, Brussels, Belgium; 2 Laboratory of Experimental Surgery, Université Libre de Bruxelles, Brussels, Belgium; 3 Department of Physical Education and Rehabilitation, Université catholique de Louvain, Louvain-la-Neuve, Belgium

## Abstract

**Background:**

There are only few data relating the metabolic consequences of feeding diets very low in n-3 fatty acids. This experiment carried out in mice aims at studying the impact of dietary n-3 polyunsaturated fatty acids (PUFA) depletion on hepatic metabolism.

**Results:**

n-3 PUFA depletion leads to a significant decrease in body weight despite a similar caloric intake or adipose tissue weight. n-3 PUFA depleted mice exhibit hypercholesterolemia (total, HDL, and LDL cholesterol) as well as an increase in hepatic cholesteryl ester and triglycerides content. Fatty acid pattern is profoundly modified in hepatic phospholipids and triglycerides. The decrease in tissue n-3/n-6 PUFA ratio correlates with steatosis. Hepatic mRNA content of key factors involved in lipid metabolism suggest a decreased lipogenesis (SREBP-1c, FAS, PPARγ), and an increased β-oxidation (CPT1, PPARα and PGC1α) without modification of fatty acid esterification (DGAT2, GPAT1), secretion (MTTP) or intracellular transport (L-FABP). Histological analysis reveals alterations of liver morphology, which can not be explained by inflammatory or oxidative stress. However, several proteins involved in the unfolded protein response are decreased in depleted mice.

**Conclusion:**

n-3 PUFA depletion leads to important metabolic alterations in murine liver. Steatosis occurs through a mechanism independent of the shift between β-oxidation and lipogenesis. Moreover, long term n-3 PUFA depletion decreases the expression of factors involved in the unfolded protein response, suggesting a lower protection against endoplasmic reticulum stress in hepatocytes upon n-3 PUFA deficiency.

## Background

Dietary n-3 polyunsaturated fatty acids (PUFA) have important metabolic effects namely through their involvement in eicosanoid biosynthesis and their ability to modulate the transcription of regulatory genes [1-4]. n-3 PUFA are able to coordinate an upregulation of lipid oxidation and a downregulation of lipid synthesis [5-7]. Through their capacity to function as ligand activators of peroxisome proliferator-activated receptor α (PPARα), n-3 PUFA increase fatty acid oxidation [8,9]. On the other hand, n-3 PUFA suppress lipogenesis by inhibition of sterol regulatory element binding protein-1c (SREBP-1c) gene expression and/or proteolytic release [5]. Peroxidation of PUFA has also been proposed as a mechanism involved in the regulation of lipid metabolism, but this remains controversial [10-13].

Promoting n-3 PUFA consumption seems to be interesting in the context of reducing metabolic disorders associated to obesity. Several studies have reported the influence of n-3 PUFA supplementation on inflammation and lipid and glucose metabolism [14]. Their results give evidence of the beneficial effects of these fatty acids on triglyceridemia [7], blood pressure [15], inflammation [16,17] and insulin sensitivity [18,19]. The rationale to propose dietary supplementation with n-3 PUFA is also based on the fact that obese people have a lower level of n-3 PUFA in plasma [20,21], liver and erythrocyte phospholipids (PL) [22].

Up till now, it has been rather difficult to assess the pathophysiological relevance of the modifications of tissue fatty acid composition due to a lower n-3 PUFA intake. To clarify this point, a rat model with n-3 fatty acids depletion during two generations has bee developed. Recent data have shown that these rats display several features of the metabolic syndrome including visceral obesity [23], hepatic steatosis [24], insulin resistance [25], cardiac hypertrophy [26] and perturbation of metabolic, ionic and functional events in pancreatic islets [25,27]. The biochemical mechanism explaining such metabolic features remains unclear. The fatty acid pattern is important to take into account in order to interpret the relevance of dietary intervention focused on the physiological role of fatty acids [28]. We have pointed out that feeding a diet low in n-3 PUFA profoundly modifies the n-3/n-6 PUFA ratio in liver tissue. The modification of the hepatic fatty acid composition also changes the expression of genes considered as metabolic targets regulated by PUFA in the liver. We show that n-3 PUFA depletion is associated with hepatic triglyceride accumulation. Analysis of liver mRNA content of key factors involved in lipid metabolism demonstrates a decrease in lipogenic gene expression, and an increase in mRNA coding for enzymes/factors involved in hepatic catabolism (CPT1, PGC1α). Disturbances of the mechanism involved in cell protection, namely a decrease in the unfolded protein response, occur in hepatocytes upon prolonged n-3 deficiency and may contribute to hepatic morphological alterations.

## Methods

### Animals and diets

Control (CT) and second generation n-3 PUFA depleted (low n-3) female C57Bl/6J mice (Laboratory of Experimental Surgery, Université Libre de Bruxelles, Brussels, Belgium) were housed in groups of four mice per cage (twelve per group) at 22°C in an 12 h light/dark cycle and were given free access to diet and water. The control diet (AO3, SAFE, Villemoison-sur-orge, France) contained the following (percent w/w): protein 21, total carbohydrate 52 (including starch 34, cellulose 4), soya oil 5, vitamin and mineral mixture 5 and water 12. The low n-3 diet contained (percent; w/w) casein 23, corn starch 36, saccharose 26, sunflower oil 5, agar-agar 2, cellulose 2, vitamin mixture 5 and mineral mixture 1. The n-6/n-3 ratio was 6.1 and 12.4 for the control diet and the low n-3 diet, respectively. The proportion of the n-3 fatty acids as a percentage of total fatty acids was 9% in the control diet and less than 2% in the low n-3 diet. The relative proportion of n-6 fatty acids was 55% in the control diet and 22% in the low n-3 diet. The decrease in total PUFA in the low n-3 diet was compensated by an increase in the proportion of monounsaturated fatty acids (MUFA) (+ 17%) and saturated fatty acids (+14%). The major n-3 and n-6 PUFA in the diets were α-linolenic acid and linoleic acid, respectively. There were only traces of long chain PUFA (EPA, DHA...) in both control and low n-3 diet. The detailed fatty acid pattern of these diets was fully described previously [25].

All mice experiments were approved by the local animal ethics committee and the housing conditions were as specified by the Belgian Law of November 14, 1993 on the protection of laboratory animals (agreement n° LA 1230314).

### Food intake assessment

Food intake, taking into account spillage, was recorded twice weekly during the last three weeks as previously described [29,30]

### Oral glucose tolerance test

An oral glucose tolerance test (gavage with 3 mg glucose/g body weight; 66% glucose solution) was performed on 6h-fasted mice one week before the end of the treatment. Blood glucose was determined with a glucose meter (Roche diagnostic) on 3.5 μl of blood collected from the tip of the tail vein, 30 min before and 0, 15, 30, 60, 90 and 120 min following glucose injection. Insulin was measured in 5 μl of plasma samples obtained from tail blood at -30 and 15 min using an ELISA kit (Mercodia, Upssala, Sweden).

### Tissue and blood samples

At the age of 34 ± 1 weeks, mice were anaesthetized by intra-peritoneal injection of sodium pentobarbital solution (Nembutal^®^, 60 mg/kg of body weight, Sanofi Santé Animale, Benelux, Brussels). Vena cava blood samples were collected in EDTA tubes. After centrifugation (10 min at 1500 g), plasma was stored at -80°C. A fraction of the main liver lobe was fixed-frozen in isopentane and kept at -80°C for histological analysis. The excess tissue material was immediately clamped in liquid N_2 _and kept at -80°C. Full and empty caecum, liver, spleen and fat tissues (ovarian, subcutaneous, and visceral) were collected and weighed.

### Liver histological analysis

For the morphological analysis, liver sections were stained with hematoxylin-eosin. For the detection of neutral lipids, frozen sections were sliced and stained with the oil red O, using 0.5% oil red O dissolved in propylene glycol for 10 min at 60°C. The sliced sections were then counterstained.

### Blood biochemical analysis

Plasma triglycerides (TG), cholesterol (Elitech diagnostics, Sees, France), β-hydroxybutyrate (Stanbio Laboratory, Boerne, USA) and non esterified fatty acid (NEFA) (Wako, Neuss, Germany) concentrations were measured using kits coupling enzymatic reaction and spectrophotometric detection of reaction end-products. High density lipoprotein cholesterol (HDL-C) (Diasys Diagnostic and Systems, Holzheim, Germany) concentration was measured enzymatically after very low density lipoprotein (VLDL), chylomicrons and low density lipoprotein cholesterol (LDL-C) antibodies precipitation. LDL was estimated by the Friedwald formula [31]. Cytokines were determined in 12 μl of plasma using a kit (Bio-Plex Multiplex; Bio-Rad, Nazareth, Belgium) and measured using Luminex technology (Bio-Plex; Bio-Rad).

### Tissue biochemical analysis

Fatty acid content was determined in tissue PL and TG as reported before [32]. For hepatic lipid content measurement, one gram of liver tissue was homogenized in 10 ml of phosphate buffer (pH 7.4). Lipids were extracted by mixing 125 μl of homogenate with 1 ml of 2:1 chloroform: methanol (Folch et al. 1957). The chloroform phase was evaporated under nitrogen flux, and the dried residue was solubilized in 100 μl of isopropanol. TG or cholesterol were measured as previously described for plasma samples. Free cholesterol (Diasys Diagnostic and Systems, Holzheim, Germany) was determined using a kit coupling enzymatic reaction and spectrophotometric detection of reaction end-products. Peroxidation was evaluated by measuring liver thiobarbituric acid-reactive substance content. Aldehydes contained in tissue homogenates reacted with thiobarbiuric acid forming an aldehyde-TBA complex, which can be spectrophotometrically detected [33]. Hepatic glycogen content was assessed as follows: 20 mg of tissue were dissolved in NaOH 1 M at 55°C for 1 h, neutralized with HCl 1 M and centrifuged. An aliquot of the supernatant was incubated in the presence of amyloglucosidase (Merck, Darmstadt, Germany) for 2 h at 37°C in a shaking bath. Free glucose was measured as previously described for plasma samples.

### SDS/PAGE and immunoblotting

Approximately 30 mg of frozen liver were homogenized in RIPA buffer (50 mM HCl, 150 mM NaCl, 1 mM EDTA, 1% NP-40, 0.25% deoxycholic acid, 2 mM sodium orthovanadate, 5 mM phenylmethylsulfonyl fluoride and a protease inhibitor cocktail). The homogenates were then centrifuged for 20 min at 13,000 g. Cell lysates (30 μg) were combined with Laemmli sample buffer and separated by SDS/PAGE. After electrophoretic separation at 40 mA, the proteins were transferred to a PVDF membrane at 80 V for 2 h followed by western blot analysis. Membranes were then incubated in a 5% Blotto solution. Subsequently, membranes were incubated overnight at 4°C with the following antibodies diluted (1:1000) in TBST (tris-buffered saline tween-20) containing 1% BSA (bovine serum albumin): BIP (binding protein), PDI (protein disulfide isomerase), MBTPS2 (membrane-bound transcription factor peptidase, site 2), IRE1α (inositol-requiring enzyme 1 alpha), p-PERK [PKR (double-stranded RNA-activated protein kinase R)-like ER kinase], total PERK, p-JNK (c-jun N-terminal kinase) and total JNK. All antibodies were purchased from Cell Signaling except total PERK (Abcam, Cambridge, UK). Membranes were washed in TBST and incubated for 1 h at room temperature in a secondary antibody conjugated to horseradish peroxidase (1:10,000, Cell Signaling). After additional washes, chemiluminescence detection was carried out using an Enhanced Chemiluminescent Western blotting kit (ECL Plus, Amersham Biosciences) and hyperfilms (Hyperfilm ECL, Amersham Biosciences). Then, the membranes were stripped and re-probed with an antibody recognizing GAPDH (Abcam) to which all data were reported. The films were scanned with an ImageScanner using the Labscan software and quantified with the Image Master 1D Image Analysis Software (Amersham Biosciences).

### Real-time quantitative PCR

Total RNA was isolated from liver tissue (Roche Diagnostics Belgium, Vilvoorde). cDNA was prepared by reverse transcription of 1 μg total RNA using the Kit Reverse transcription System (Promega, Leiden, The Netherlands). Real-time PCRs were performed with the GeneAmp 5700 sequence detection system and software (Applied Biosystems, Den Ijssel, The Netherlands) using SYBER-Green for detection. RPL19 RNA was chosen as an invariant standard. Primers and gene details are summarized in table 1. All tissues were run in duplicate in a single 96-well reaction plate (MicroAmp Optical, Applied Biosystems) and data were analysed according to the 2^-ΔACCT ^method. The identity and purity of the amplified product were checked through analysis of the melting curve carried out at the end of amplification.

**Table 1:** Sequences for the primers used in real-time quantitative PCR

	GenBank accession no.	Forward primer (5' to 3')	Reverse primer (5' to 3')
PGC1α	NM_008904.1	AGCCGTGACCACTGACAACGAG	GCTGCATGGTTCTGAGTGCTAAG
CPT1a	NM_013495.1	AGACCGTGAGGAACTCAAACCTAT	TGAAGAGTCGCTCCCACT
PPARα	NM_011144.3	CAACGGCGTCGAAGACAAA	TGACGGTCTCCACGGACAT
PPARγ	NM_011146.2	CTGCTCAAGTATGGTGTCCATGA	TGAGATGAGGACTCCATCTTTATTCA
FAS	NM_007988.3	TTCCAAGACGAAAATGATGC	AATTGTGGGATCAGGAGAGC
SREBP-1c	NM_011480.2	GATCAAAGAGGAGCCAGTGC	TAGATGGTGGCTGCTGAGTG
NOX	NM_007807.2	TTGGGTCAGCACTGGCTCTG	TGGCGGTGTGCAGTGCTATC
MTTP	NM_008642.1	ATGATCCTCTTGGCAGTGCTT	TGAGAGGCCAGTTGTGTGAC
DGAT2	NM_026384.3	ACTCTGGAGGTTGGCACCAT	GGGTGTGGCTCAGGAGGAT
GPAT1	NM_008149.3	GTCCTGCGCTATCATGTCCA	GGATTCCCTGCCTGTGTCTG
G6Pase	NM_008061.3	AGGAAGGATGGAGGAAGGAA	TGGAACCAGATGGGAAAGAG
PEPCK	NM_011044.2	ACCTCCTGGAAGAACAAGGA	CTCATGGCTGCTCCTACAAA
L-FABP	NM_017399.2	ACCTCATCCAGAAAGGGAAGG	ACAATGTCGCCCAATGTCATG

PGC1α; peroxisome proliferator-activated receptor gamma coactivator, CPT1; carnitine palmitoyl transferase 1, PPAR; peroxisome proliferator-activated receptor, FAS; fatty acid synthase, SREBP-1c; sterol-regulatory-element-binding protein-1c, NOX; NADPH oxidase, MTTP; microsomal triglycerides transfert protein, DGAT2; Diacylglycerol acyl transferase 2, GPAT1; Glycerol phosphate acyl transferase 1, G6Pase; glucose 6-phosphatase, PEPCK; phosphoenolpyruvate carboxykinase, L-FABP; liver fatty acid binding protein

### Statistical analysis

Results are presented as mean ± SEM. Statistical significance between groups was assessed by Student *t*-test using GraphPad Prism version 4.00 for Windows. Pearson's correlation test was used to estimate correlation between two parameters. *P *< 0.05 was considered as statistically significant.

## Results

### Food intake, body weight and organ weights

Daily energy intake was monitored during the last three weeks of the experiments. No difference was detected between CT and low n-3 mice (10.09 ± 0.66 and 9.22 ± 0.05 kcal/cage x day, respectively). Table 2 shows that body, liver and spleen weights were statistically significantly lower in low n-3 mice compared to CT mice. There was not significant effect of dietary manipulation on visceral, subcutaneous, and ovarian adipose tissues. Both caecal tissue and caecal content weights were significantly reduced by 59% and 56%, respectively, in low n-3 mice.

**Table 2:** Body and tissue weight

	**CT**	**Low n-3**
Body weight (g)	22.6 ± 0.7	20.7 ± 0.4*
Liver (g/100 g body wt)	4.50 ± 0.15	3.97 ± 0.08*
Spleen (g/100 g body wt)	0.51 ± 0.06	0.37 ± 0.03*
Pancreas (g/100 g body wt)	0.67 ± 0.04	0.72 ± 0.04
Visceral adipose tissue (g/100 g body wt)	0.66 ± 0.11	0.66 ± 0.02
Ovarian adipose tissue (g/100 g body wt)	0.81 ± 0.14	1.02 ± 0.08
Sub-cutaneous adipose tissue (g/100 g body wt)	1.21 ± 0.16	1.41 ± 0.06
Caecal tissue (g/100 g body wt)	0.58 ± 0.03	0.33 ± 0.02*
Caecal content (g)	0.17 ± 0.01	0.10 ± 0.01*

Data are mean ± SEM. *: means significantly different from the CT group (P < 0.05, Student *t*-test). n ≥ 8 per group.

### Parameters related to glucose metabolism (Table 3)

**Table 3:** Glucose metabolism

	**CT**	**Low n-3**
Fasting serum glucose (mmol/l)	5.20 ± 0.29	6.09 ± 0.27*
Fasting serum insulin (pmol/l)	86.2 ± 13.3	92.6 ± 10.8
		
Post-OGTT serum glucose (mmol/l)	14.5 ± 0.6	16.7 ± 0.9
Post-OGTT serum insulin (pmol/l)	90.4 ± 27.1	217.5 ± 20.2*
		
Hepatic glycogen content (μg/mg prot)	70.9 ± 15.6	103.7 ± 21.0

OGTT: serum was obtained 15 min after an oral glucose tolerance test. Data are mean ± SEM. *: means significantly different from the CT group (P < 0.05, Student *t*-test). n ≥ 8 per group.

Low n-3 mice exhibited a higher fasting glycaemia compared to CT mice with no effect on fasting insulinemia. Fifteen minutes after oral glucose loading, plasma insulin concentrations were strongly increased in low n-3 mice but their glucose response was similar (AUC mM.min over 120 min: CT 1851 ± 205, low n-3 1705 ± 130). The liver glycogen content was higher in low n-3 mice but this was not statistically significant (p = 0.2). No difference was detected between CT and low n-3 mice in liver mRNA coding gluconeogenic key enzymes: phosphoenolpyruvate carboxykinase (relative expression, CT = 1.00 ± 0.14, low n-3 = 1.07 ± 0.16) and glucose 6-phosphatase (relative expression, CT = 1.00 ± 0.27, low n-3 = 1.31 ± 0.25).

### Blood markers of lipid homeostasis

**Figure 1:**
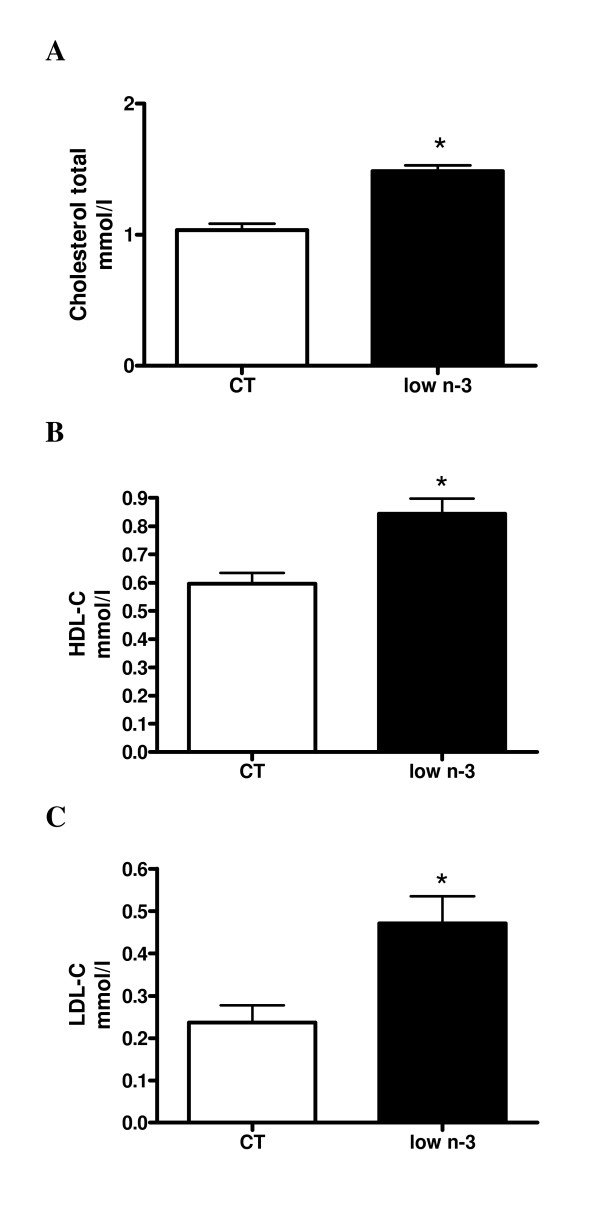
**Cholesterol concentration**. (A) Total cholesterol, (B) HDL-C and (C) LDL-C were measured in the plasma at the end of the experiment. Data are mean ± SEM. *: means significantly different from the CT group (P < 0.05, Student *t*-test). n ≥ 8 per group.

β-OHbutyrate, NEFA and TG levels, measured in the vena cava, were not modified by the dietary manipulation (β-OHbutyrate in mM: CT 0.14 ± 0.02, low n-3 0.14 ± 0.02; NEFA in mM: CT 0.22 ± 0.04, low n-3 0.26 ± 0.04; TG in mM: CT 0.41 ± 0.04, low n-3 0.34 ± 0.04). However, low n-3 mice had higher plasma total, HDL, and LDL-cholesterol concentrations (Figure 1).

### Liver fatty acid pattern (Table 4)

**Table 4:** Fatty acid pattern in liver triglycerides and phospholipids fractions

	**Triglycerides**		**Phospholipids**	
**(μg/g liver)**	**CT**	**Low n-3**	**CT**	**Low n-3**

C12:0	3.44 ± 3.34	7.88 ± 4.51	ND	ND
C14:0	130.7 ± 24.4	242.6 ± 34.3*	22.0 ± 0.9	20.6 ± 0.7
C16:0	4509 ± 360	10320 ± 1054*	4955 ± 77	4718 ± 220
C18:0	395.2 ± 128.1	587.4 ± 76.0	3255 ± 74	3984 ± 203*
C20:0	6.06 ± 3.39	21.7 ± 1.0*	20.9 ± 3.6	17.2 ± 0.5
C22:0	ND	ND	70.8 ± 15.5	98.9 ± 5.4*
C24:0	ND	ND	90.9 ± 5.9	105.5 ± 2.5
				
C16:1 n-7	895.2 ± 39.9	1302 ± 201	307.0 ± 20.4	176.9 ± 10.0*
C18:1 n-9	8188 ± 662	16780 ± 2151*	1828 ± 105	1860 ± 146
C20:1 n-9	140.7 ± 17.6	239.4 ± 24.2*	52.6 ± 5.3	39.7 ± 3.9
				
C18:3 n-3	ND	ND	ND	ND
C20:5 n-3	467.0 ± 9.0	ND	205.4 ± 21.4	ND
C22:3 n-3	ND	ND	ND	ND
C22:5 n-3	87.7 ± 24.4	ND	138.2 ± 7.2	36.0 ± 3.2*
C22:6 n-3	757.5 ± 121.4	50.5 ± 5.1*	4705 ± 132	675.2 ± 48.5*
				
C18:2 n-6	3540 ± 394	12414 ± 1563*	3644 ± 72	3859 ± 208
C18:3 n-6	58.2 ± 13.9	296.6 ± 64.1*	44.7 ± 4.9	63.0 ± 7.1
C20:2 n-6	41.6 ± 2.4	72.0 ± 5*	86.9 ± 9.6	34.3 ± 10.6*
C20:3 n-6	ND	ND	ND	ND
C20:4 n-6	271.0 ± 40.7	813.6 ± 204.3*	4581 ± 82	7665 ± 228*
C22:4 n-6	55.0 ± 6.4	142.9 ± 41.1	59.3 ± 3.7	169.9 ± 10.0*

Data are mean ± SEM. *: means significantly different from the CT group (P < 0.05, Student *t*-test). n ≥ 8 per group.

The dietary manipulation differently affected the liver fatty acid pattern in PL and in TG (Table 4). A two-fold increase in saturated fatty acids (myristic acid C14:0, palmitic acid C:16:0 and arachidic acid C20:0) and monounsaturated fatty acids (oleic acid C18:1 n-9 and eicosenoic acid C20:1 n-9) was observed in liver TG of low n-3 mice. Only minor changes in saturated fatty acid pattern of the PL fraction were observed.

Alpha-linolenic acid C18:3 n-3 was undetectable in liver PL and TG fractions of either CT or low n-3 mice. All long chain n-3 derivatives were lower in both liver PL and TG fractions of low n-3 mice. In low n-3 mice docosahexaenoic acid C22:6 n-3 decreased drastically to reach 15% of the control value in liver PL fraction, and 7% of the control value in liver TG fraction. Eicosapentaenoic acid C20:5 n-3 was not detectable in liver PL and TG fractions of low n-3 mice. Arachidonic acid C20:4 n-6 doubled in liver PL fraction of low n-3 mice compared to CT mice, whereas linoleic acid C18:2 n-6 content was not modified in PL. In liver TG fraction of low n-3 animals, there was a three fold increase in both linoleic and arachidonic acid levels.

### Liver lipid metabolism

**Figure 2:**
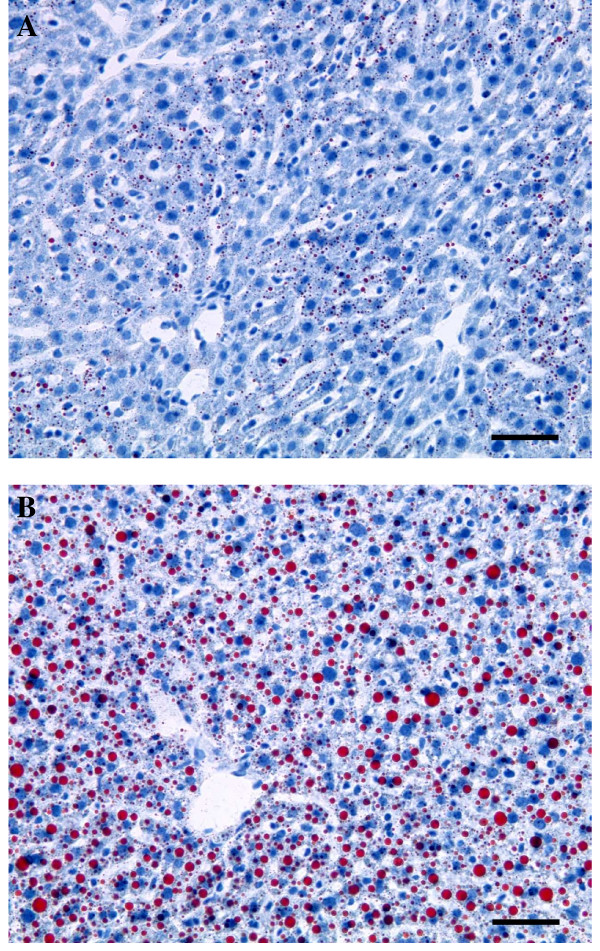
**Fat staining of liver section**. (A) CT mice and (B) Low n-3 mice. Oil red staining was performed on frozen section. Bar = 50 μm.

**Figure 3:**
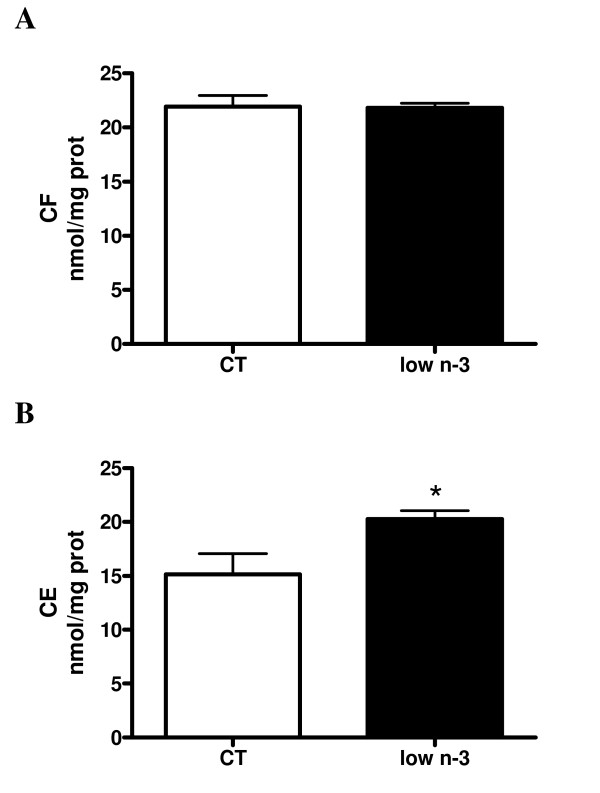
**Liver cholesterol content**. CF: free cholesterol; CE: esterified cholesterol. Data are mean ± SEM. *: means significantly different from the CT group (P < 0.05, Student *t*-test). n ≥ 8 per group.

**Figure 4:**
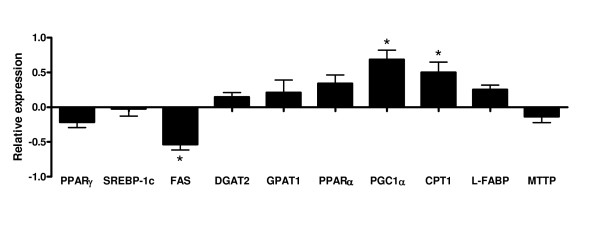
**Liver gene expression**. The data are presented as a relative increase (positive value) or decrease (negative value) of mRNA liver content measured in low n-3 mice versus CT (set at 0 value). See table 1 for primer sequences and abbreviations. Data are mean ± SEM. *: means significantly different from the CT group (P < 0.05, Student *t*-test). n ≥ 8 per group.

Histological analysis of low n-3 mice liver revealed higher liver macro vesicular TG content (Fig 2). This steatosis was confirmed by biochemical analysis (TG nmol/mg proteins: CT 97.6 ± 10.0, low n-3 159.0 ± 53.4, p = 0.006). The amount of TG in liver tissue positively correlated with the n-6/n-3 ratio in both PL (r^2 ^= 0.97; p = 0.0001) and TG (r^2 ^= 0.87; p = 0.0007) fractions. A slight increase in total cholesterol was observed in low n-3 mice (nmol/mg proteins: CT = 37.06 ± 2.56, low n-3 = 42.09 ± 0.81, p = 0,067), which reflected a significant increase in the esterified fraction (Fig 3). The excess of lipids deposited in the liver can theoretically result from increased uptake of circulating lipids, from enhanced de novo lipogenesis, from decreased fatty acid oxidation, or from decreased hepatic lipoprotein secretion. The key enzymes and nuclear factors involved in the control of those metabolic pathways were analysed at the mRNA level. PPARγ is a lipogenic transcription factor who has already been associated with steatosis [34]. No modification of its expression occurred in low n-3 mice. Likewise no change was detected in SREBP-1c expression, another lipogenic factor under partial control of insulin [35,36,36]. Low n-3 mice exhibited a lower fatty acid synthase (FAS) expression, the rate-limiting enzyme for fatty acids synthesis (Fig 4). No effect in low n-3 mice was observed for diacylglycerol acyl transferase 2 (DGAT2), which is a key enzyme for the esterification of diacylglycerol to TG, and on glycerol phosphate acyl transferase 1 (GPAT1), which is the mitochondrial enzyme catalysing the first step of fatty acid esterification to TG and PL. The expression of transcription factors involved in fatty acid oxidation, namely peroxisome proliferator-activated receptor gamma coactivator α (PGC1α) and PPARα (p = 0.069), was increased in low n-3 mice. Carnitine palmitoyl transferase 1 (CPT1), the rate limiting enzyme for mitochondrial β-oxidation, shows a higher expression in low n-3 mice compared to CT (p = 0.052) (Fig 4). These results suggest a lower lipogenic enzyme activity and a higher hepatic β-oxidation capacity in low n-3 mice. Liver fatty acid binding protein (L-FABP), which regulates hepatic fatty acids trafficking, was not modified. The expression of microsomal triglyceride transfer protein (MTTP), which drives the association of lipids to apoprotein B100 to allow formation of VLDL and further export, was unchanged (Fig 4).

### Liver histological analysis

**Figure 5:**
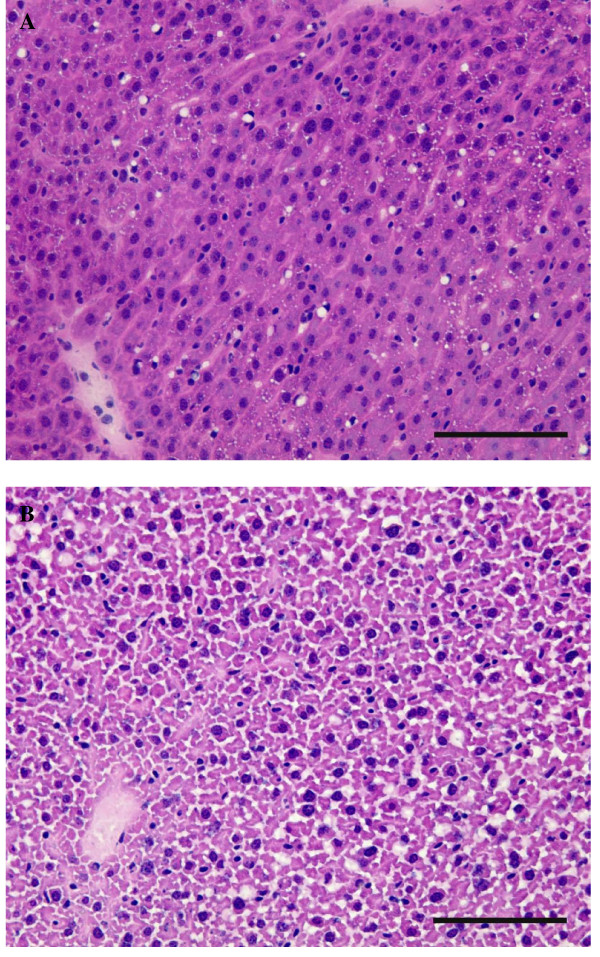
**Histological analysis of liver section**. (A) CT mice and (B) Low n-3 mice. Hematoxylin and eosin staining; bar = 100 μm.

Histological analysis revealed alterations in hepatic tissue structure of low n-3 mice. Hepatocytes were shrunk and nuclei were abnormally dense. No necrotic foci or leukocyte infiltration were observed. Compared to low n-3 mice, stellate cells were more visible in CT mice (Fig 5).

#### Parameters reflecting cellular stress in liver tissue

**Figure 6:**
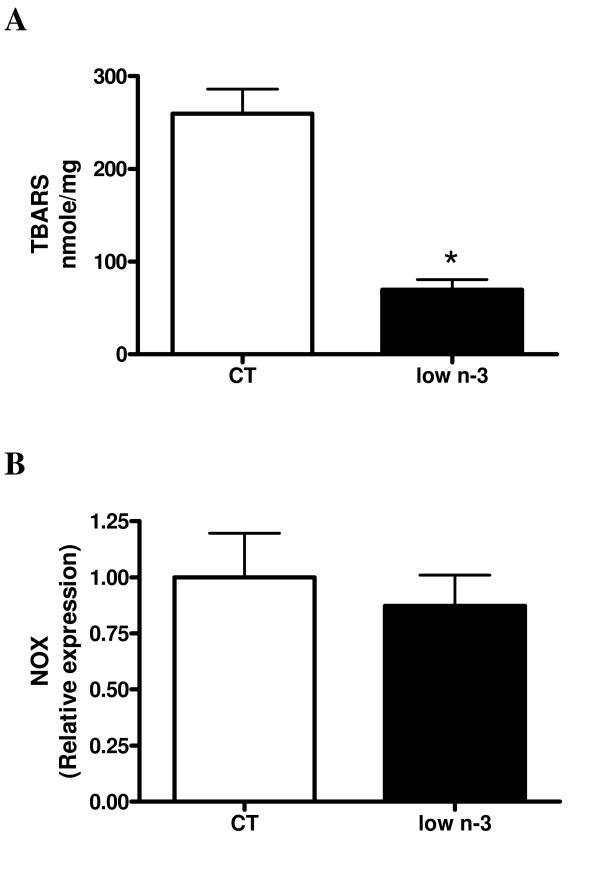
**Liver oxidative stress**. (A) TBARS (thiobarbituric acid-reactive substance) content in liver tissue. (B) NOX (NADPH oxidase) mRNA level in hepatic tissue. Data are mean ± SEM. *: means significantly different from the CT group (P < 0.05, Student *t*-test). n ≥ 8 per group.

Several markers of cellular stress were analysed in liver tissue. Figure 6 shows a paradoxical lower liver lipid peroxide content in low n-3 mice compared to CT mice, but no difference in the expression of NADPH oxidase. Different cytokines (monocyte chemoattractant protein-1, tumor necrosis factor α, interleukin 1β, interleukin 6) were measured in the vena cava but no difference in concentration was detected between groups (data not shown).

**Figure 7:**
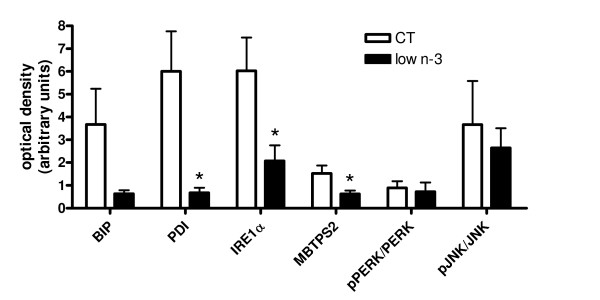
**Liver proteins involved in the unfolded protein response**. The amount of protein was measured for BIP (binding protein), PDI (protein disulfide isomerase), IRE1α (inositol-requiring enzyme 1 alpha) and MBTPS2 (membrane-bound transcription factor peptidase, site 2) after optical density scanning of the western blot incubated with the corresponding antibody, as specified in the material and methods section. Phosphorylated PERK [PKR (double-stranded RNA-activated protein kinase R)-like ER kinase] and phosphorylated JNK (c-jun N-terminal kinase) were reported to their respective total form. Data are mean ± SEM. *: means significantly different from the CT group (P < 0.05, Student t-test). n ≥ 8 per group.

Low n-3 mice exhibited a significant lower hepatic content of several proteins involved in the unfolded protein response (UPR): PDI, IRE1α and MBTPS2. Likewise, BIP was lower in liver of low n-3 mice but the difference did not reach statistical significance (p = 0.07). No difference was observed between CT and low n-3 mice for the ratios of phosphorylated PERK and phosphorylated JNK to their respective total form (Fig 7).

## Discussion

The objective of this study was to investigate the metabolic and toxicologic consequences of n-3 PUFA depletion through a decrease in n-3 PUFA dietary intake for two generations in mice with a special emphasis on liver tissue. The fatty acid pattern analysis confirmed the n-3 PUFA depletion in hepatic PL and TG fractions of mice fed for a prolonged period with a diet characterized by low n-3 PUFA content. Moreover, low n-3 mice exhibited an increase in saturated, monounsaturated and polyunsaturated n-6 fatty acids mainly in the hepatic TG fraction.

Liver total cholesterol was slightly higher in low n-3 mice, and this was mainly due to an increased esterified cholesterol fraction. Cholesterolemia (total, HDL and LDL) was increased in low n-3 mice. The higher MUFA content in the liver PL and TG fractions of low n-3 mice could be responsible for the increased cholesterol esterification. As a matter of fact, the enzyme involved in cholesterol esterification, acyl-Coenzyme A: cholesterol acyltransferase 2 (ACAT2), preferentially uses MUFA to other fatty acids [37]. Moreover, previous studies confirm that oleoyl-CoA and palmitoleyl-CoA liver content, which are increased in low n-3 mice hepatic TG, are crucial to synthesize esterified cholesterol [38].

Total TG were significantly higher in liver tissue of low n-3 mice compared to CT mice. Steatosis was confirmed both biochemically and histologically in low n-3 mice.

The abnormal accumulation of TG in liver tissue had already been shown in rats before [24] but, in that rat model, it was related to an increase in fat mass development [23]. Here, low n-3 mice developed hepatic steatosis, despite a lower body weight gain and a similar adipose tissue weight as compared to CT mice, thus suggesting that steatosis is always present in n-3 deficiency, independent of fat mass accumulation.

To analyse the biochemical mechanism involved in TG accumulation, the expression of several factors involved in lipid metabolism was evaluated in liver tissue of low n-3 mice as compared to CT mice. Among the factors involved in the control of hepatic steatosis by PUFA, PPARα has been considered important[39], even if recent data are controversial [40]. The liver of low n-3 mice showed a higher expression of CPT1, which is compatible with a higher amount of two cofactors involved in oxidative pathways: PGC1α and PPARα mRNA. Werner et al. suggested that PPARα activation can be driven by non-essential LCPUFA, namely the n-9 and n-7 fatty acid families [41]. In fact, we have shown that these fatty acids were increased in the liver PL and TG fractions of low n-3 mice compared to CT mice, which could lead to PPARα dependent gene expression.

Low n-3 mice exhibited a lower hepatic expression of FAS, the rate-limiting enzyme for fatty acid synthesis, whereas SREBP-1c and PPARγ mRNA levels, considered as drivers of FAS expression and often associated with tissue TG accumulation [34,35], were not modified. Insulin is a well known inducer of fatty acid synthesis, acting through SREBP-1c activation [35]. In fact, low n-3 mice showed a higher fasting serum glycaemia compared to CT mice. This can not be explained through a higher expression of key gluconeogenic enzymes (phosphoenolpyruvate carboxykinase and glucose-6-phosphatase). Moreover, 15 min after a glucose load, low n-3 mice showed a higher insulin secretion. Therefore, it appears that the liver of low n-3 animals, despite a higher exposure to glucose and insulin – reflected by a higher hepatic glycogen content – directs fatty acids towards catabolic pathways rather than towards anabolic pathways. This is also supported by the lack of effect of n-3 PUFA depletion on the expression of enzymes controlling PL and TG synthesis (GPAT1 and DGAT2 expression). L-FABP and MTTP involved, respectively, in the intracellular transport of fatty acids and in the export of TG were not modified by n-3 depletion. Therefore, the abnormal accumulation of TG in the liver tissue could be a consequence of hepatic injury rather than the result of specific metabolic disturbances associated with n-3 deficiency.

In fact, the histological analysis revealed liver morphological alterations in low n-3 mice compared to CT mice. To explain this hepatic injury, several markers of stress were measured. No difference was found in the inflammatory and oxidative stress between CT and low n-3 mice. The endoplasmic reticulum stress was also evaluated. A recent study in rats suggests that the composition of fatty acids in steatotic liver is an important determinant of susceptibility to liver injury [42]. It was shown that hepatic steatosis characterized by increased saturated fatty acids leads to liver injury, endoplasmic reticulum (ER) stress and impaired regenerative response to liver injury. Moreover, some models of ER stress show decreased hepatic TG secretion [43] which may worsen steatosis. ER stress is caused by the accumulation of unfolded proteins and protein aggregates in the ER lumen [44]. To maintain ER function when the secretory pathway is compromised, cells have developed an adaptive mechanism called the unfolded protein response. There are three proximal sensors: PERK, ATF6 and IRE1α. When phosphorylated, PERK leads to a general decrease of protein translation. Like IRE1α, ATF6 is involved in the ER chaperone gene transcriptional induction. IRE1α is also responsible for the degradation of aggregated protein and the JNK phosphorylation. BIP is a chaperone protein and PDI is a folding catalysts [44,45]. MBTPS2 catalyses the ATF6 cleavage necessary for its activity [46]. Curiously, western blot analysis revealed a decrease in most of these proteins involved in the unfolded protein response (UPR) namely PDI, MBTPS2, and IRE1α. On the contrary, the hepatic content of phospho-PERK/PERK and phospho-JNK/JNK was similar between low n-3 and CT mice. IRE1α knockout fibroblasts or mice show no alteration in the transcriptional ER chaperone induction [47,48]. However, MBTPS2 deletion inhibits ATF6 cleavage and consequently fails to induce BIP [46]. Moreover, MBTPS2 cleavage seems also to be necessary for the transcriptional induction of IRE1α [47]. Therefore, the lower MBTPS2 content may be the key event explaining the decrease in UPR proteins. These results suggest that long term n-3 PUFA depletion leads to a loss in content of proteins related to ER stress, which are classically considered as a mechanism of protection against cell injury. Therefore, we propose that this mechanism is disturbed by changes in tissue n-3 PUFA levels, thus could contributing to morphological alterations of liver tissue. The link with steatosis remains unclear.

Alternatively, mitochondrial dysfunction is another stress characterized by hepatic steatosis. It has been shown that mice deficient in long-chain acyl-CoA dehydrogenase exhibited higher hepatic lipid storage, increased cholesterolemia without any modification in triglyceridemia and no change in CPT1, PPARα, SREBP-1c, ACC or DGAT2 expression [49]. The similarities in the metabolic phenotype of low n-3 mice and mice lacking long-chain-acyl-CoA dehydrogenase suggest that mitochondrial dysfunction could be an interesting target to study. Moreover, there are indications suggesting that n-3 depletion in the mitochondrial membrane is associated with impaired oxidation [50].

## Conclusion

In conclusion, n-3 PUFA depletion in liver tissue promotes steatosis, and leads to alterations in the mechanisms of protection of liver tissue against unfolded protein induced stress.

The biochemical mechanism involved in TG accumulation remains unknown, and a dynamic study to analyse VLDL secretion and/or mitochondrial stress by liver tissue could be interesting perspectives for further studies. Our results suggest that the lack of dietary n-3 fatty acids – observed e.g. in obese and diabetic people can be part of hepatotoxic events linked to steatosis.

## Abbreviations

BIP: binding protein; CPT1: carnitine palmitoyl transferase 1; DGAT2: Diacylglycerol acyl transferase 2; ER: endoplasmic reticulum; FAS: fatty acid synthase; GPAT1; Glycerol phosphate acyl transferase 1; HDL-c: high density lipoprotein-cholesterol; IRE1α: inositol-requiring enzyme 1 alpha; JNK: c-jun N-terminal kinase; LDL-c: low density lipoprotein-cholesterol; L-FABP: liver fatty acid binding protein; MBTPS2: membrane-bound transcription factor peptidase, site 2; MTTP: microsomal triglycerides transfert protein; MUFA: monounsaturated fatty acid; NEFA: non esterified fatty acid; PDI: protein disulfide isomerase; PERK: PKR (double-stranded RNA-activated protein kinase R)-like ER kinase; PGC1α: peroxisome proliferator-activated receptor gamma coactivator α; PL: phospholipid; PPARα or γ: peroxisome proliferator-activated receptor α or γ; PUFA: polyunsaturated fatty acid; SREBP-1c: sterol regulatory element binding protein-1c; TG: triglyceride; UPR: unfolded protein response; VLDL: very low density lipoprotein.

## Authors' contributions

PBD designed the study, carried out all experiments and dosages (except the hepatic fatty acid profile and the western blot), analysed the data and wrote the manuscript. NAM and CPD conceived the study, performed the statistical analysis of the data and revised the manuscript. PL, MWJ and CYA performed the hepatic fatty acid pattern analysis and participated in the discussion of the results. DL and FM performed the western blots and participated to the discussion of the results. DBF, SFM participated in the blood and tissue sampling and preparation, as well as to some biochemical measurements. BLB performed cytokine measurements. DNM conceived and designed the study, interpreted the results, coordinated the experiments, helped to draft the manuscript and critically reviewed and revised its final version. All authors read and approved the final manuscript.
